# Isolation and Characterization of Antibodies Induced by Immunization with TNF-α Inducible Globotetraosylceramide

**DOI:** 10.3390/ijms21103632

**Published:** 2020-05-21

**Authors:** Tetsuya Okuda

**Affiliations:** Bioproduction Research Institute, National Institute of Advanced Industrial Science and Technology (AIST), Tsukuba 305-8566, Japan; t-okuda@aist.go.jp; Tel.: +81-50-3648-6162

**Keywords:** antibody, globoside/Gb4Cer, Gb3Cer, VLCFA, TNF-α, hybridoma

## Abstract

Glycosphingolipids containing very-long-chain fatty acids (VLCFAs) regulate several immune responses, such as cytokine production, immune signaling, and antibody induction. We previously reported that stimulation with an inflammatory mediator, TNF-α, promotes the expression of glycosphingolipids in vascular endothelial cells. The major component is globotetraosylceramide containing VLCFAs (Gb4Cer-VLCFAs), but its role in inflammatory responses has not been fully investigated. In this study, the antibody-inducing properties of Gb4Cer-VLCFAs were analyzed using serum and hybridoma cells generated from Gb4Cer-VLCFA-immunized mice. The reactivity of serum antibodies against Gb4Cer indicated that immunization with Gb4Cer-VLCFAs immediately induced the production of anti-Gb4Cer antibodies. Over 81% of hybridomas generated from the splenocytes of an immunized mouse produced anti-Gb4Cer antibodies, a subset of which recognized an epitope shared by Gb4Cer and its precursor globotriaosylceramide (Gb3Cer). Further biochemical analyses of established monoclonal antibodies revealed that these antibodies included IgM and IgG3, which specifically react with Gb4Cer and Gb3Cer. These results indicate that immunization with Gb4Cer-VLCFAs can efficiently induce the production of anti-Gb4Cer and -Gb3Cer antibodies by B cells.

## 1. Introduction

Glycosphingolipids (GSLs) are cell membrane components composed of oligosaccharides and ceramides. Oligosaccharides and ceramides in GSLs are structurally diverse, and recent studies have revealed that GSLs containing very-long-chain fatty acids (VLCFAs) in the ceramide portion are involved in immune responses in mammalian tissues [1,2,3,4]. For example, α-linked monosaccharyl ceramides such as α-galactosylceramide, which is isolated from the marine sponge *Agelas mauritianus*, contain VLCFAs in the ceramide portion [1] that activate mammalian natural killer T (NKT) cells [1,5] and promote cytokine production in a VLCFA-dependent manner [1,2]. GSLs containing VLCFAs also play an important role in the association of Src family kinases with membrane microdomains in neutrophils, which mediates signal transduction associated with neutrophil migration and phagocytosis [3]. The stimulation of vascular endothelial cells (ECs) with lipopolysaccharide (LPS) activates Toll-like receptor signaling, resulting in inflammatory responses in the ECs. LPS stimulation also promotes the production of GSLs containing VLCFAs by ECs, and these molecules are thought to reduce excess EC inflammatory responses via the inhibition of inflammatory signaling [4].

We previously reported that the stimulation of human umbilical vein ECs with an inflammatory mediator, TNF-α, promotes GSL production in ECs via the transcriptional regulation of genes related to GSL synthesis [6]. Further structural analyses revealed that the primary component of these GSLs is globotetraosylceramide/globoside (Gb4Cer) containing VLCFAs (Gb4Cer-VLCFAs) [6,7]. To characterize the function of these GSLs in ECs, we generated anti-Gb4Cer antibodies and found that Gb4Cer-VLCFAs exhibits efficient antibody-inducing activity in mice. The immunization of mice with Gb4Cer-VLCFAs immediately induced the production of serum antibodies that specifically reacted with Gb4Cer. Analyses of hybridoma cells generated from splenocytes isolated from a mouse immunized with Gb4Cer-VLCFAs revealed that hybridoma clones producing the anti-Gb4Cer antibodies could be easily isolated, and that these clones produced both IgM- and IgG-class antibodies. Furthermore, some of these antibodies reacted with both Gb4Cer and its precursor, globotriaosylceramide (Gb3Cer), indicating that these antibodies recognize a shared epitope in these GSLs. These results indicate that Gb4Cer-VLCFAs function as immunity inducers for the production of anti-Gb4Cer and -Gb3Cer antibodies in mice.

## 2. Results

### 2.1. Induction of Anti-Gb4Cer Antibodies in Serum from Mice Immunized with Gb4Cer-VLCFAs

**Figure 1:**
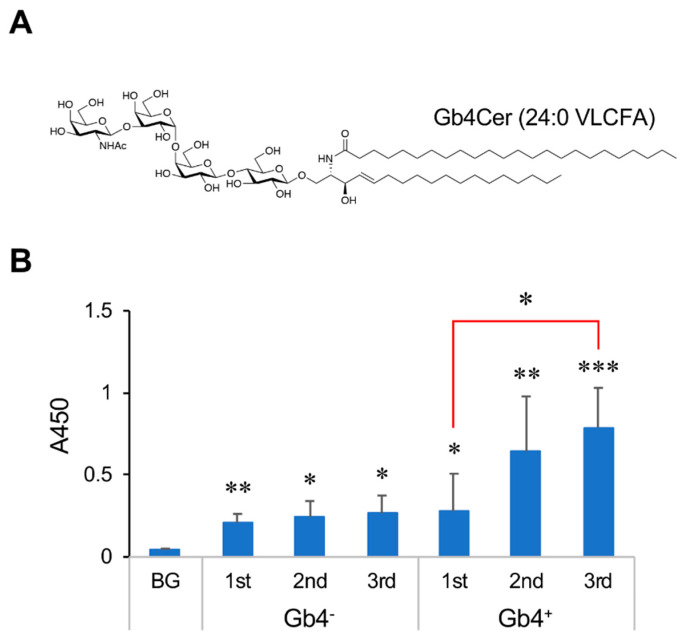
Reactivity of the serum of mice immunized with globotetraosylceramide containing very-long-chain fatty acids (Gb4Cer-VLCFAs) against Gb4Cer. (**A**) Chemical structure of Gb4Cer containing C24:0 VLCFA. (**B**) Mice were immunized with Gb4Cer-VLCFAs using a liposome method as described in *Materials and Methods*, and serum samples prepared 3 days after the first subcutaneous immunization (1st) and the second (2nd) and third (3rd) intraperitoneal immunizations were analyzed by enzyme-linked immunosorbent assay (ELISA). BG, serum from un-treated mice; Gb4^+^, serum from mice immunized with Gb4Cer-containing liposomes; Gb4^−^, serum from mice immunized with Gb4Cer-free liposomes. Error bars: mean ± S.D. (*n* = 3–5). * *p* < 0.05, ** *p* < 0.01, *** *p* < 0.001 BG vs. each serum or 1st vs. 3rd (red line).

The stimulation of ECs with TNF-α promotes the production of Gb4Cer, which primarily contains C24:0 VLCFA (Figure 1A), via the transcriptional regulation of genes related to Gb4Cer-VLCFAs synthesis [6,7]. As the ceramide portion of Gb4Cer from human erythrocytes predominantly contains C24:0 VLCFA [7], we used Gb4Cer-VLCFAs in immunization experiments. Gb4Cer-VLCFAs also contain C22:0, C22:1, C24:1, and C26:1 VLCFAs as subcomponents [7]. Mice were immunized with Gb4Cer-VLCFAs using a liposome method [8], and the reactivity of total serum immunoglobulins against Gb4Cer was analyzed by enzyme-linked immunosorbent assay (ELISA) (Figure 1B).

Immunization with Gb4Cer-VLCFAs significantly increased the reactivity of serum antibodies against Gb4Cer, and booster immunizations after 2 weeks further significantly enhanced this reactivity (Figure 1B, Gb4+). The reactivity of the serum against Gb4Cer was also slightly increased in control mice immunized with Gb4Cer-VLCFA-free liposomes (Figure 1B, Gb4−), but this was unchanged after booster immunization. These results indicate that the repetitive immunization of mice with Gb4Cer-VLCFAs efficiently increases the population of B cells producing anti-Gb4Cer antibodies.

### 2.2. Characterization of Antibodies Produced by Hybridoma Cells Generated from a Gb4Cer-VLCFA-Immunized Mouse

**Figure 2:**
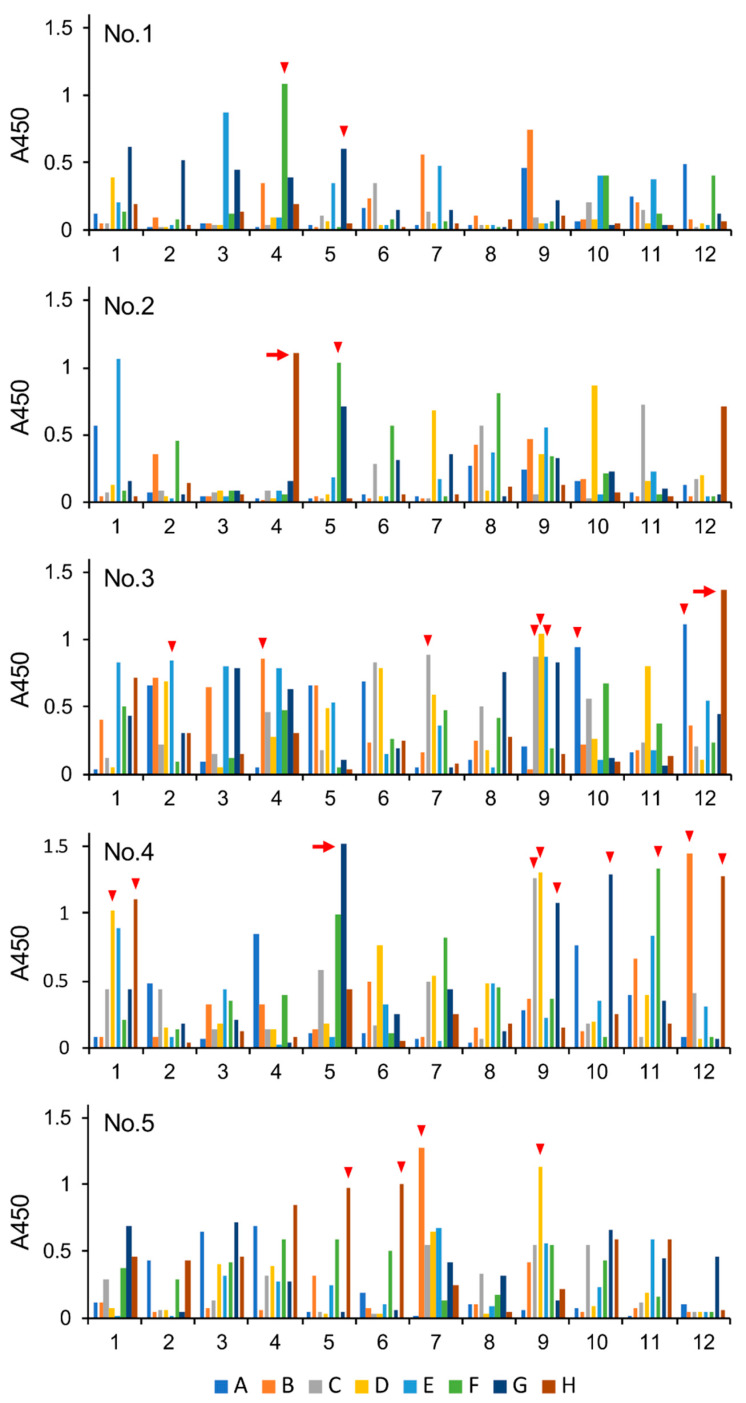
Reactivity of hybridoma culture supernatants against Gb4Cer. Hybridoma cells prepared from a Gb4Cer-VLCFA-immunized mouse were seeded into five 96-well microplates to grow as a single colony per well, and the reactivity of the antibodies in the supernatant of each well against Gb4Cer was analyzed by ELISA using horseradish peroxidase-labeled anti-mouse Immunoglobulin G (H + L). A through H and 1 through 12 indicate the row and well numbers of the 96-well plate. These results are summarized in Table 1. Arrows and arrowheads correspond to the clones analyzed in Figure 3. Arrows correspond to the clones established as hybridomas as follows: 4G5, PA5; 3H12, PA4.2; 2H4, PA7.

To characterize the antibodies produced by B cells in Gb4Cer-VLCFA-immunized mice, we isolated lymphocytes from the spleen and generated hybridoma cells. The spleen was isolated from a mouse in which the serum exhibited an average level of reactivity against Gb4Cer among the immunized mice we examined. The hybridomas were seeded into 96-well microtiter plates to grow as a single colony per well, and the reactivity against Gb4Cer of antibodies in the supernatant of each well was analyzed by ELISA (Figure 2).

**Figure 3:**
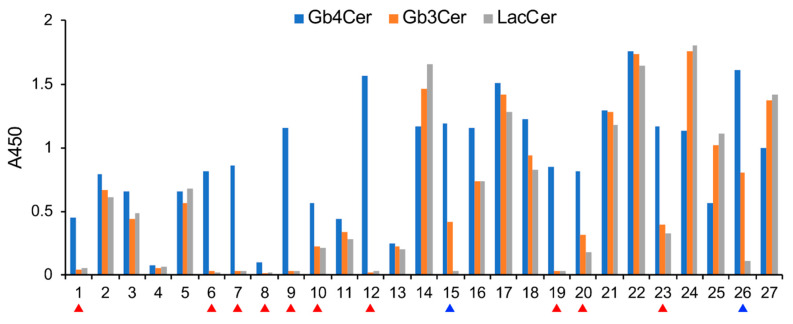
Epitope specificity of antibodies in hybridoma culture supernatants. The reactivity against Gb4Cer, globotriaosylceramide (Gb3Cer), and lactosylceramide (LacCer) of antibodies in hybridoma culture supernatants from 27 clones showing strong reactivity to Gb4Cer in Figure 2 was analyzed by ELISA. These results are summarized in Table 2. Red and blue arrowheads correspond to clones producing anti-Gb4Cer and anti-Gb4Cer/Gb3Cer antibodies, respectively.

In this experiment, supernatants from 480 wells were analyzed, and >81% of the hybridomas produced antibodies that reacted with Gb4Cer (Table 1). Over 63.5% of these hybridomas produced anti-Gb4Cer antibodies with moderate reactivity, whereas 20.2% of the hybridomas produced antibodies that strongly reacted with Gb4Cer. Then we selected 27 clones from the 20.2% of hybridomas that produced strongly reacting antibodies and analyzed their specificity using the Gb4Cer precursors Gb3Cer and lactosylceramide (LacCer) (Figure 3).

A total of 37% of the selected hybridomas produced antibodies that specifically reacted with Gb4Cer, and 7.4% of these hybridomas produced antibodies that reacted with both Gb4Cer and its precursor, Gb3Cer (Table 2). The remaining hybridomas produced low-specificity antibodies that reacted with Gb4Cer, Gb3Cer, and their precursor LacCer. Additional ELISAs using horseradish peroxidase-labeled anti-mouse IgM revealed that almost all of the antibodies that specifically reacted with Gb4Cer were of the IgM class. Further analyses using an antibody isotyping kit indicated that one clone (Figure 3, No. 9) produced anti-Gb4Cer IgG3(κ). All of the antibodies that reacted with Gb4Cer and Gb3Cer were IgM.

### 2.3. Properties of the Anti-Gb4Cer Monoclonal Antibodies Isolated in This Study

To characterize the specificity of these monoclonal antibodies (mAbs) in detail, we isolated three clones from the hybridomas that produced antibodies that specifically and strongly reacted with Gb4Cer. Using the culture supernatants of these clones, we determined the epitope of each mAb using ELISA. GSLs with similar oligosaccharide structures in Gb4Cer, described in Table 3, were used for this analysis.

**Figure 4:**
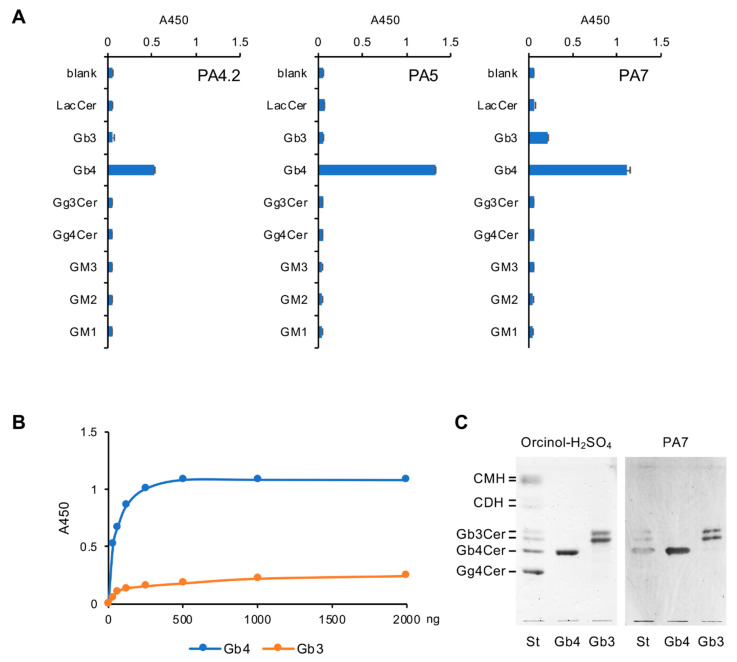
Characterization of monoclonal antibodies generated from a Gb4Cer-VLCFA-immunized mouse. (**A**) Reactivity of monoclonal antibodies (mAbs) PA4.2, PA5, and PA7 against several GSLs, evaluated by ELISA. The wells of a 96-well microtiter plate were coated with 500 ng of each GSL and then reacted with each mAb at a concentration of 2 μg/mL. The structures of the GSLs are summarized in Table 3. (**B**) Reactivity of mAb PA7 against various concentrations of Gb4Cer (Gb4) and Gb3Cer (Gb3), evaluated by ELISA. (**C**) Thin-layer chromatography (TLC)-immunostaining of Gb4Cer using PA7. The double band found in Gb3Cer is due to the difference in the lipid structure. Left panel, orcinol-H_2_SO_4_ staining. Right panel, immunostaining with PA7. St, standard glycosphingolipids mixture; Gb4, Gb4Cer; Gb3, Gb3Cer; CMH, ceramide monohexoside; CDH, ceramide dihexoside.

Figure 4A shows that mAb IgG3(κ) and IgM(κ), produced by hybridoma clones PA4.2 and PA5 respectively, specifically reacted with Gb4Cer but not with other similar GSLs. Other properties of the PA4.2 and PA5 mAbs have been reported elsewhere [9,10]. Hybridoma clone PA7 produced an IgM(κ) mAb that specifically reacted with both Gb4Cer and Gb3Cer. Although previous studies described several mAbs that specifically react with Gb4Cer [11,12], this is the first report of a mAb that recognizes an epitope shared by Gb4Cer and Gb3Cer. Further ELISAs using the PA7 mAb and 96-well plates coated with various concentrations of Gb4Cer or Gb3Cer indicated that the PA7 mAb reacts with each GSL in a dose-dependent manner with high reactivity (Figure 4B). Although the PA7 mAb exhibited weaker reactivity against Gb3Cer than Gb4Cer, this mAb was suitable for thin-layer chromatography (TLC)-immunostaining for the structural analysis of Gb3Cer as well as Gb4Cer (Figure 4C). These results indicate that the unique immune-inducing activity of Gb4Cer-VLCFAs stimulates the production of a variety of antibodies that specifically react with Gb4Cer and Gb3Cer in mice.

## 3. Discussion

The results of this study demonstrate that antibodies that recognize Gb4Cer and its precursor Gb3Cer can be easily produced by immunizing mice with Gb4Cer-VLCFAs. The antibodies produced include IgM and IgG3, indicating that Gb4Cer-VLCFAs induce class switching in B cells. The ability to induce class switching is associated with T-cell-independent type-type 2 polysaccharide antigens that induce potent B-cell responses [13]. Although previous studies described several mAbs that specifically react with Gb4Cer [9,11,12], no IgG-class mAbs were isolated. Furthermore, the PA7 mAb isolated in the present study reacted with an epitope shared by Gb4Cer and Gb3Cer. Such unique specificity has not been observed in previously described antibodies that react with Gb4Cer or similar GSLs.

As oligosaccharides are generally less immunogenic than proteins, it is difficult to induce animals to generate antibodies that specifically recognize glycoconjugates. For example, a previous study [14] in which hybridomas were isolated from a mouse immunized with a glycolipid containing a mammal-derived oligosaccharide reported that only 3% of the hybridomas produced antibodies that reacted with the oligosaccharide. All of these hybridomas produced IgM, and only one clone isolated from all 518 hybridomas analyzed produced a mAb that specifically recognized the oligosaccharide as an epitope. In the case of Gb4Cer-VLCFAs, the frequency of hybridomas producing anti-Gb4Cer was much higher in this case than that previously reported (Table 1 and Table 2).

In contrast, Figure 1B shows that the immunization of mice with Gb4Cer-VLCFA-free liposomes resulted in increased reactivity of serum antibodies against Gb4Cer. The Gb4Cer-VLCFA-free liposomes contained lipid A as an adjuvant, which can activate B cells independent of the B-cell antigen receptor. Thus, we consider that the increased reactivity of these serum antibodies against Gb4Cer was due to the production of anti-Gb4Cer antibodies by the activation of endogenous B cells with lipid A. The presence of such endogenous B cells may promote the induction of adaptive immune responses by Gb4Cer-VLCFAs. Based on these results, we conclude that immunization with Gb4Cer-VLCFAs can promote an efficient production of antibodies against Gb4Cer and Gb3Cer in mice.

Several model studies using artificial GSLs containing fatty acids of varying length support our conclusion [15,16]. These previous studies found a correlation between the length of the fatty acid in artificial GSLs and their immunogenicity, and reported that an artificial GSL containing C24:0 VLCFA exhibited potent antibody-inducing activity in mice. In addition to this artificial GSL, Gb4Cer-VLCFAs also contain C24:0 VLCFA and exhibited efficient antibody-inducing activity. These observations also suggest that the adaptive immune system of mammals can recognize VLCFAs. In the case of NKT cell activation, glycolipid antigens containing VLCFAs are recognized by cluster of differentiation 1d (CD1d) expressed by antigen-presenting cells, and the glycolipid antigen-CD1d complex stimulates NKT cells to promote cytokine production [5]. Such lipid antigen recognition molecules may also be involved in adaptive immune responses mediated by Gb4Cer-VLCFAs.

Although this study revealed that Gb4Cer-VLCFAs induces the production of antibodies that react with Gb4Cer and Gb3Cer, the immunologic role of these antibodies remains unknown. Gb4Cer and Gb3Cer are expressed on the surface of mammalian cells and function as receptors for toxins produced by enterohemorrhagic *Escherichia coli* [17,18]. However, other infectious bacteria, such as *Neisseria gonorrhoeae* and *Haemophilus influenzae*, express Gb4- and Gb3-type oligosaccharides on the cell surface [19,20,21]. As these Gram-negative bacteria also produce the inflammatory mediator LPS, infection with these organisms elicits tissue inflammation. As inflammation promotes Gb4Cer-VLCFA synthesis in the tissues, antibodies induced by Gb4Cer-VLCFAs may play a role in host defense against these microbial pathogens. Previous studies also reported that cell surface Gb4Cer is recognized by parvovirus B19, which utilizes the molecule as a receptor for entry into the cell [22]. As viral entry can be blocked by treating cells with anti-Gb4Cer antibodies [22], anti-Gb4Cer antibodies induced by Gb4Cer-VLCFAs may inhibit the interaction of cell surface GSLs with viruses and infectious bacteria.

## 4. Materials and Methods

### 4.1. Immunization and Preparation of Serum

C3H/HeN strain mice (CREA Japan, Tokyo) were used in this study. Gb4Cer from human erythrocytes (G4274, Sigma-Aldrich, St. Louis, MO, USA) was used as the Gb4Cer-VLCFA. A liposome immunization method [8] was used to immunize mice with Gb4Cer. In brief, 100 μg of Gb4Cer-VLCFAs was mixed with 10 μg of lipid A, 0.5 μmol of cholesterol, and 0.5 μmol of dipalmitoylphosphatidylcholine. The mixture was dissolved in phosphate-buffered saline (PBS) and used as an immunogen. The animals were first subcutaneously immunized, followed by intraperitoneal booster immunization 2 weeks after the first immunization. Serum was prepared from blood collected from the tail vein of the mice 3 days after each immunization. The Committee for Experiments Involving Animals of the National Institute of Advanced Industrial Science and Technology (AIST) approved all animal experiments.

### 4.2. Hybridoma Generation

The immunized mice described above were immunized an additional 3 times at 2-week intervals. Three days after the last immunization, splenocytes were collected from the mice and fused with mouse Sp2/0-Ag14 myeloma cells (RIKEN CELL BANK, Tsukuba, Japan). Hybridomas were selected using hypoxanthine-aminopterin-thymidine (HAT) selection medium: RPMI-1640 containing 10% fetal calf serum, 0.1 mM sodium hypoxanthine, 0.4 µM aminopterin, 16 µM thymidine, 10 µg/mL gentamicin, and 5% Briclone (DS Pharma Biomedical, Osaka, Japan). Culture supernatants were evaluated by ELISA, and positive clones were selected based on reactivity against Gb4Cer as an index.

### 4.3. Characterization of Immunoglobulin Isotypes

Hybridoma supernatant was diluted 50-fold with 1% bovine serum albumin in PBS, and the isotypes of antibodies present in the supernatant were determined using a mouse monoclonal antibody isotyping kit (Roche Diagnostics GmbH, Mannheim, Germany) according to the manufacturer’s instructions.

### 4.4. ELISAs

ELISAs were performed as described previously [9]. In brief, GSLs in methanol were applied to the wells of a 96-well microtiter plate and immobilized by drying. After washing twice with PBS, blocking buffer (1% bovine serum albumin in PBS) was added to each well and incubated for 15 min at room temperature, followed by the addition of diluted serum (1:100) or hybridoma culture supernatant (1:1). After 3 h of incubation at room temperature, the wells were washed with 0.1% Tween 20 in PBS, and HRP-linked anti-Ig (H + L) was added as the secondary antibody. An HRP substrate (1-Step Ultra TMB-ELISA Substrate; Pierce, Rockford, IL) was used to detect antibody binding, and the results were measured as absorbance at 450 nm. Bovine brain-derived LacCer, Gg3Cer, Gg4Cer, GM2 and GM1, and bovine milk-derived GM3 were obtained from Sigma-Aldrich. Gb3Cer from porcine erythrocytes was obtained from Nacalai Tesque, Inc. (Kyoto, Japan). 

### 4.5. TLC-Immunostaining

TLC-immunostaining was performed as described elsewhere [10]. GSLs were analyzed on HPTLC plates (Merck, Darmstadt, Germany) with a solvent system consisting of chloroform/methanol/water (60:35:8 (*v/v/v*)). Standard glycosphingolipids were visualized using orcinol-H_2_SO_4_. TLC-immunostaining was performed using PBST-diluted hybridoma supernatant containing 7.6 μg/mL of PA7 antibody. Antibody binding was detected using an ABC kit (Vector Laboratories, Burlingame, CA) and Immunostain HRP-1000 (Konica Minolta Medical and Graphic, Inc., Tokyo, Japan).

### 4.6. Statistical Analysis

After the determination of variance using the F-test, the statistical significance of differences in the data was evaluated using the two-tailed Student′s *t*-test, with statistical significance defined as * *p* < 0.05, ** *p* < 0.01, and *** *p* < 0.001.

## Figures and Tables

**Table 1:** Frequency of hybridoma clones producing anti-Gb4Cer antibodies.

Reactivity to Gb4Cer	Number of Clones	Rate (%)
Strong (A450 ≥ 0.5)	97	20.2
Moderate (0.5 > A450 ≥ 0.1)	208	43.3
Weak (0.1 > A450 ≥ 0.05)	88	18.3
Trace or negative (A450 < 0.05)	87	18.1

**Table 2:** Number of hybridoma clones producing antibodies with high reactivity to Gb4Cer and Gb3Cer.

Epitope	Number of Clones	Rate (%)
Gb4Cer	10	37.0
Gb4Cer and Gb3Cer	2	7.4
Gb4Cer and precursors	15	55.6

**Table 3:** Structures of glycosphingolipids (GSLs) used in this study.

GSL	Structure
LacCer	Galβ1,4GlcCer
Gb3Cer	Galα1,4Galβ1,4GlcCer
Gb4Cer	GalNAcβ1,3Galα1,4Galβ1,4GlcCer
Gg3Cer	GalNAcβ1,4Galβ1,4GlcCer
Gg4Cer	Galβ1,3GalNAcβ1,4Galβ1,4GlcCer
GM3	Neu5Acα2,3Galβ1,4GlcCer
GM2	GalNAcβ1,4(Neu5Acα2,3)Galβ1,4GlcCer
GM1	Galβ1,3GalNAcβ1,4(Neu5Acα2,3)Galβ1,4GlcCer

## Abbreviations

GSLs	Glycosphingolipids
Cer	Ceramide
VLCFAs	Very long-chain fatty acids
NKT cells	Natural killer T cells
ECs	Vascular endothelial cells
LPS	Lipopolysaccharide
Gb4Cer	Globotetraosylceramide/globoside
Gb3Cer	Globotriaosylceramide
LacCer	Lactosylceramide
ELISA	Enzyme-linked immunosorbent assay
mAbs	Monoclonal antibodies
TLC	Thin-layer chromatography
CD1d	Cluster of differentiation 1d
